# Prdx1 Reduces Intracerebral Hemorrhage-Induced Brain Injury via Targeting Inflammation- and Apoptosis-Related mRNA Stability

**DOI:** 10.3389/fnins.2020.00181

**Published:** 2020-03-10

**Authors:** Guo-Qiang Yang, Jia-Cheng Huang, Jun-Jie Yuan, Qin Zhang, Chang-Xiong Gong, Qiong Chen, Qi Xie, Le-Xing Xie, Ru Chen, Zhong-Ming Qiu, Kai Zhou, Rui Xu, Guo-Hui Jiang, Xiao-Yi Xiong, Qing-Wu Yang

**Affiliations:** Department of Neurology, Xinqiao Hospital, Army Medical University, Chongqing, China

**Keywords:** RNA-binding protein, peroxiredoxin 1, intracerebral hemorrhage, inflammation, apoptosis, posttranscriptional regulation

## Abstract

RNA-binding proteins (RBPs) have been shown to be involved in posttranscriptional regulation, which plays an important role in the pathophysiology of intracerebral hemorrhage (ICH). Peroxiredoxin 1 (Prdx1), an RBP, plays an important role in regulating inflammation and apoptosis. On the basis that inflammation and apoptosis may contribute to ICH-induced brain injury, in this study, we used ICH models coupled with *in vitro* experiments, to investigate the role and mechanism of Prdx1 in regulating inflammation and apoptosis by acting as an RBP after ICH. We first found that Prdx1 was significantly up-regulated in response to ICH-induced brain injury and was mainly expressed in astrocytes and microglia in ICH rat brains. After overexpressing Prdx1 by injecting adeno-associated virus (AAV) into the striatum of rats at 3 weeks, we constructed ICH models and found that Prdx1 overexpression markedly reduced inflammation and apoptosis after ICH. Furthermore, RNA immunoprecipitation combined with high-throughput sequencing (RIP-seq) *in vitro* revealed that Prdx1 affects the stability of inflammation- and apoptosis-related mRNA, resulting in the inhibition of inflammation and apoptosis. Finally, overexpression of Prdx1 significantly alleviated the symptoms and mortality of rats subjected to ICH. Our results show that Prdx1 reduces ICH-induced brain injury by targeting inflammation- and apoptosis-related mRNA stability. Prdx1 may be an improved therapeutic target for alleviating the brain injury caused by ICH.

## Introduction

Intracerebral hemorrhage (ICH) is a common type of stroke, with increasing incidence and mortality worldwide (Krishnamurthi et al., 2013). Increasing evidence shows that inflammation and apoptosis contribute to the brain injury seen in ICH, which is closely related to the severity of the patient’s symptoms and prognosis (Zheng et al., 2016). However, although the treatments of these therapeutic targets are effective in animal models (Chang et al., 2018; Fang et al., 2019), there are still no effective drugs used in the clinic (Chang et al., 2017). Therefore, improved therapeutic targets that could inhibit both inflammation and apoptosis need to be developed for the treatment of ICH.

Accessing posttranscriptional regulation is important to influence the occurrence and development of diseases, such as ICH (Dykstra-Aiello et al., 2015, 2016; Stamova et al., 2019). Posttranscriptional regulation includes various processes, such as alternative splicing (AS) (Paschalis et al., 2018), alternative polyadenylation (APA) (Tian and Manley, 2017), gene expression regulation (Roundtree et al., 2017), and RNA methylation (Zhao et al., 2017). Among the components of these regulative processes, RNA-binding proteins (RBPs) are critical for posttranscriptional regulation because they can influence the other regulative processes by binding to RNA and because they play vital roles in RNA modification (Hentze et al., 2018). Moreover, posttranscriptional regulation can be controlled via RBPs (Zhang et al., 2015; Fei et al., 2017). Therefore, targeting posttranscriptional regulation via RBPs may alleviate the brain injury caused by ICH, as the progression of ICH has been shown to be closely regulated by posttranscriptional regulation (Dykstra-Aiello et al., 2015, 2016; Stamova et al., 2019).

Although Prdx1, an RBP (Baltz et al., 2012; Castello et al., 2012; Kim et al., 2012; Kwon et al., 2013; Sebestyén et al., 2016), has been shown to be elevated after ICH (Nakaso et al., 2000), its roles in brain injury after ICH are still largely unknown. Prdx1 is a member of the peroxiredoxin family (Wood et al., 2003) and has been shown to be involved in regulating many pathological processes, such as redox reactions (Yamaguchi et al., 1993), inflammatory responses, apoptosis (Liu et al., 2014, 2018; Min et al., 2018; Lu et al., 2019), and tumorigenesis (Hoshino et al., 2005; Cao et al., 2009). These findings suggest that Prdx1 may also play important roles in regulating the above-mentioned pathological processes to influence the brain injury caused by ICH.

In this study, we thus created ICH models and coupled them with *in vitro* experiments to investigate the role that Prdx1 might play in regulating inflammation and apoptosis by acting as an RBP as well as the underlying mechanism. We found that elevated expression of Prdx1 can significantly reduce inflammation and apoptosis after ICH, and that the protective effects of Prdx1 against brain injury may be related to the binding of inflammation- and apoptosis-related mRNAs, as revealed by RIP-seq technology.

## Materials and Methods

### Animals

Male Sprague-Dawley (SD) rats (8–10 weeks old, 250–300 g) were purchased from the Animal Center of Daping Hospital, Third Medical Military University, Chongqing, China. All rats were raised in a clean environment and maintained at 25 ± 2°C under a 12 h light/12 h dark cycle and free access to food and water. All procedures and animal experiments were performed in agreement with the Provision and General Recommendation of Chinese Experimental Animals Administration Legislation and the Animal Management Committee of the Third Military Medical University.

### ICH Model

The ICH model was established as described in previous studies (Xu et al., 2019). Briefly, autologous blood (70 μL) without anticoagulant or the same volume of saline was injected into the right striatum (0.2 mm anterior and 3.5 mm lateral of bregma at a depth of 5.5 mm) with a syringe pump (KD Scientific, Holliston, MA, United States), at a rate of 10 μL/min. The needle was left in for 10 min after the injection and then slowly withdrawn, and the skin was sutured. The success rate of the model was 90%; failed models and dead rats were excluded from this study.

### Construction of the Prdx1 Overexpression Rat Model

The Prdx1-overexpressed adeno-associated virus (AAV2/9-r-Prdx1-3 × flag-mCherry) was named Prdx1-OE-AAV, and vector (AAV2/9-CMV-mCherry) was purchased from Hanbio Biotechnology (Hanbio, China). Five microliters (1.2 × 1012 vg/mL) of Prdx1-OE-AAV or the same volume of vector was injected at 1 μL/min into the right striatum (0.2 mm anterior and 3.5 mm lateral of bregma at a depth of 5.5 mm). Three weeks after the operation, the ICH model was established. The expression effect of the virus was verified by western blot analysis and quantitative real-time PCR (qRT-PCR).

### Immunofluorescence Staining

As described in our previous report (Xiong et al., 2016), the brain tissue was fixed with 4% paraformaldehyde. After gradient dehydration, the tissue was embedded and cut into 30 μm thick sections. The sections were permeated at 37°C for 1 h with 0.5% Triton-X-100, blocked with 5% BSA for 2 h, and then incubated with the following primary antibodies overnight at 4°C: anti-NeuN (1:500, Abcam, Cambridge, United Kingdom), anti-GFAP (1:200, Abcam), anti-Iba1(1:200, Abcam) and anti-Prdx1 (1:200, Abcam). The sections were then incubated with fluorescent secondary antibody for 2 h at 37°C. Fluorescent secondary antibodies including Alexa Fluor 647 (1:200, donkey anti-goat), Alexa Fluor 647 (1:200, donkey anti-mouse), and Alexa Fluor 488 (1:200, donkey anti-rabbit), were all obtained from Invitrogen (Carlsbad, CA, United States). Next, 4′,6-diamidino-2-phenylindole (DAPI, 1:3000) was applied for 10 min, and samples were rinsed in PBS. All images were captured using a confocal fluorescence microscope (Leica TCS Sp5, Mannheim, Germany). Number of positive cells were counted using ImageJ software and analyzed in three different arbitrary units that can be defined as the average number of positive cells.

### Immunohistochemistry Staining

Following our previous methods (Fang et al., 2014), the brain tissue was fixed with 4% paraformaldehyde, embedded in paraffin and cut into 3.5 μm thick sections. The tissues were dewaxed in xylene, rehydrated in alcohol, placed in 100°C sodium citrate buffer for antigen retrieval for 20 min, and immersed in 3% hydrogen peroxide at 37°C for 10 min to block peroxidase activity. The sections were blocked with 5% BSA for 2 h, and then incubated with the anti-Prdx1 (1:200, Abcam) overnight at 4°C. The sections were then incubated with a rabbit polyclonal secondary antibody (Maravai LifeSciences, San Diego, CA, United States) at 37°C for 2 h, incubated with SABC (Maravai) at 37°C for 1 h, and the color reaction was developed with diaminobenzidine (Zsbio, Beijing, China). All images were captured using a light microscope (BX51, Olympus, Tokyo, Japan). Numbers of positive cells were calculated using ImageJ software and analyzed in three different arbitrary units that can be defined as the average number of positive cells.

### Quantitative Real-Time PCR

Using our previously described method to isolate total RNA and perform qRT-PCR (Meng et al., 2017), tissue and cellular RNA were extracted using TRIzol (Invitrogen, Carlsbad, CA) and RNA was reverse transcribed into cDNA (Takara, Dalian, China). Quantitative RT-PCR was performed on an ABI PCR instrument (ABI, CA, United States) using iQ SYBR Green reagent. Primers were synthesized by Shanghai Shenggong Biotechnology Co., Ltd. The primer sequences used in this study are shown in Supplementary Table 1. Relative gene expression levels were determined using the 2^–ΔΔCT^ method.

### Western Blot Analyses

As described in our previous report (Wang et al., 2014), protein was extracted and separated on 12% SDS-PAGE (Beyotime Biotechnology, Shanghai, China), and then transferred onto polyvinylidene fluoride membranes (Merck Millipore, Temecula, CA, United States) by electroblotting. The membranes were blocked with 5% BSA for 2 h at room temperature and incubated with the following primary antibodies overnight at 4°C: prdx1 (1:1000, Bosterbio, Pleasanton, CA, United States), Bcl2 (1:1000, Abcam), Bax (1:1000, Abcam), and β-actin (1:1000, Santa Cruz Biotechnology, Dallas, TX, United States). The membranes were washed with TBS-T washing buffer and incubated with HRP-conjugated goat anti-rabbit secondary antibodies (1:10000, Zsbio, Beijing, China) or anti-mouse secondary antibodies (1:10000, Zsbio) at 25°C for 2 h. Bound antibodies were visualized using an enhanced chemiluminescence (ECL) substrate and gray values were evaluated with ImageJ software.

### Brain Water Content

We demonstrated in our previous study (Xiong et al., 2016) that the brain water content (BWC) reflects the degree of brain edema. The rats were anesthetized by intraperitoneal injection with pentobarbital, and cerebral tissues were removed, and the hematoma and contralateral brain tissue were preserved by heating to 100°C for 24 h. The BWC was calculated using the formula ((wet weight – dry weight)/wet weight) × 100%.

### Nissl and Fluoro-Jade B (FJB) Staining

We performed Nissl staining as previously described (Li Q. et al., 2018). Briefly, the tissues were dewaxed in xylene, rehydrated in alcohol, incubated with Nissl staining solution (Beyotime) at 62°C for 1 h and rinsed in PBS. The procedure also followed our previously reported method (Wang et al., 2018). Tissue was adhered to the slide and rinsed in PBS, soaked in a solution containing 1% sodium hydroxide in 80% alcohol for 5 min, and then transferred to 0.06% potassium permanganate for 10 min. The samples were incubated with Fluoro-Jade B (FJB) dye (Chemicon International, Temecula, CA, United States) at 37°C for 30 min. All images were captured using a light microscope (BX51, Olympus, Japan). The number of positive cells were calculated by ImageJ software and analyzed in three different arbitrary units that can be defined as the average number of positive cells.

### RNA Immunoprecipitation, High-Throughput Sequencing (RIP-seq), and Data Analysis

The cell sample was suspended in a cell culture dish with precooled PBS, and the dish was irradiated with a UV cross-linker. Collected cells were added to the lysate at a ratio of 1:10 to lyse and digest the DNA. The lysate was incubated with the RIP antibodies overnight at 4°C for immunoprecipitation. The magnetic beads were resuspended with the antigen-antibody complex solution for 1 h, and the magnetic beads resuspended in MNase were placed on a hot mixer for 10 min and eluted. After removal of MNase, the sample was dephosphorylated with FastAP enzyme, treated with T4 PNK enzyme, and then treated with proteinase K to digest protein, and the RNA was extracted. The Illumina ScriptSeqTM v2 RNA-Seq Library Preparation Kit (Li et al., 2017; Song et al., 2018) was used for the library construction and sequencing. The quality of the RIP library was judged according to the mapping result. Quality-qualified data were entered for the downstream analysis to obtain valid reads for the genomic location distribution, peak calling, and motif analysis, which revealed the type and pattern of mRNA and ncRNA that Prdx1 binds at the genome-wide level.

### Construction of cDNA Library and Data Analysis

The DNA was digested by RQ1 DNase (Promega) enzyme in total RNA. A total of 5 μg of total RNA was taken for the polyA library construction. The PCR product with a fragment size of 300–500 bp was selected for sequencing. The results of the library were sequenced using an Illumina HiSeq X Ten sequencing platform for sequencing at 150 bp. The systematic evaluation of the data included the extraction and quality assessment of effective reads, the length distribution of effective reads, sequencing saturation statistics, and base content statistics. Differentially expressed genes (DEGs) were identified in different samples using the edgeR software system (Pan et al., 2017; Sun et al., 2017) and submitted to a DAVID function GO analysis.

### Survival Analysis

Following methods described in the literature (Meng et al., 2017), the number of dead rats was counted once every hour after ICH. To calculate the survival rate, the following equation was used: (number of ICH rats per group – number of dead rats per group)/number of ICH rats per group. A Kaplan-Meier survival analysis was then conducted to evaluate survival rates.

### Flow Cytometry

An Annexin V/Dead Cell kit was purchased from BD Biosciences (San Jose, CA, United States), and apoptosis staining was performed according to the manufacturer’s instruction. The cells were divided into normal cells, early apoptosis cells, late apoptosis cells, and dead cells using flow cytometry, and the proportion of the four types of cells was calculated.

### Hematoma Measurement

According to our previous methods (Meng et al., 2017), the rats were sacrificed at 3 d after ICH, and brain tissues were taken and frozen at −20°C for 30 min. Coronal sections of the brain were made with a thickness of 1mm. The area of hematoma in each section was determined using Image-Pro Plus 5.0 image processing software (Media Cybernetics, Bethesda, MD, United States) and the hematoma volume was measured according to the formula *V* = *t* × (*A1* + *A2* + ⋯ + *An*), where *V* is the hematoma volume, *t* is the slice thickness, and *A* is the hematoma area.

### Modified Neurological Severity Score

The modified neurological severity score (mNSS) was calculated according to previous methods (Li et al., 2019), and the detailed scoring rules are shown in Supplementary Table 2. The score is based on an evaluation of the neurological defects, according to aspects of movement, sensation, balance, etc. Higher scores indicate more obvious neurological defects. The score in the current study were determined by three experienced laboratory researchers who were unaware of the group assignments of the rat. The final score for each rat was the average of all scores for that rat.

### Primary Astrocyte Sorting

SD rats aged 8–12 weeks were anesthetized with 1% pentobarbital and perfused with pre-cooled PBS. The brain was isolated, then the brain tissue was cut into pieces of 1–3 mm with surgical scissors, and 4 mg/mL Papain(Worthington, Lakewood, United States) was added to digest the brain tissues at 37°C for 1 h; the tissues were slightly shaken every 5 min, followed by repeated pipetting of the cell suspension; then, the supernatant was used for centrifugation and cells were deposited at the bottom of the tube, using Alexa Fluor 488 GFAP antibody (1:20, BD Biosciences) combined with flow sorting to separate astrocytes for RIP.

### Experimental Design

**FIGURE 1:**
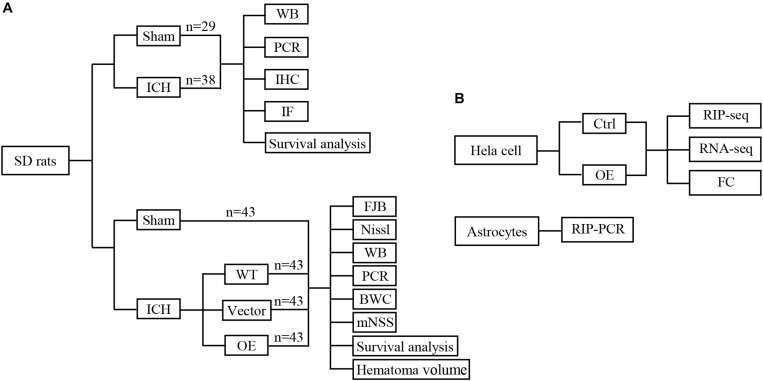
Conceptual illustrations of the experimental design. Flow diagram of *in vivo* **(A)** and *in vitro* **(B)** experiments.

First, 67 rats were randomly divided into two groups: a sham group and an ICH group, 20 rats of each group were used to calculate mortality and sacrificed at 5 days after ICH. 12 ICH rats were sacrificed at 12, 24, 72, and 120 h, respectively (*n* = 3 each timepoint), and RNA were extracted to detect the Prdx1 mRNA level. Three rats in each group sacrificed at 72 h for immunofluorescence and immunohistochemistry staining, and three rats in each group sacrificed at 72 h for western blotting (Figure 1A). Second, 132 rats were randomly divided into four groups (*n* = 43 each group): a sham group and three ICH groups (WT, Vector, Prdx1-OE); three rats of each group were used for qRT-PCR and western blotting, three rats of each group were used for FJB and Nissl staining, six rats of each group were used to detect BWC, five rats of each group were used for functional assessments, and six rats of each group were used to calculate hematoma volume, these rats all sacrificed at 3 days after ICH. Twenty rats of each group were used to calculate mortality and sacrificed at 5 days after ICH (Figure 1A and Supplementary Figures S3A,B). Finally, for the *in vitro* experiments, Prdx1-overexpressing plasmids were transfected into HeLa cells, Prdx1-overexpressing HeLa cell and the cells from control group were collected for RIP-seq, RNA-seq and flow cytometry analysis. The primary astrocytes were extracted and subjected to RIP-qRT-PCR, to verify the RIP-seq data (Figure 1B).

### Statistical Analysis

All data are expressed as the mean ± SEM or percentage. Student’s *t*-tests were used to evaluate the differences between independent samples, and comparisons among multiple groups were examined using one-way ANOVA. Two-way ANOVA was used to evaluate the differences in BWC between groups. A Kaplan Meier survival analysis was applied to evaluate survival rates. Differences were considered to be significant at *P* < 0.05.

## Results

### Prdx1 Expression Was Significantly Upregulated After ICH

**FIGURE 2:**
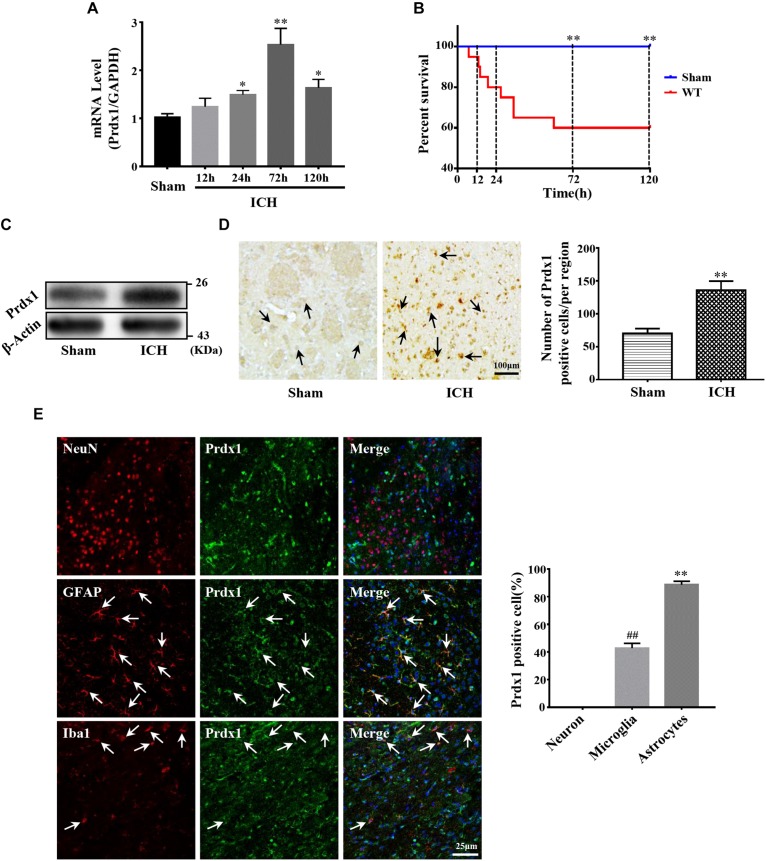
Prdx1 expression was significantly increased in rats after ICH. **(A)** Prdx1 expression in the perihematomal area was detected by qRT-PCR (df = 4, *F* = 35.945, ^∗^*P* < 0.05 versus sham, ^∗∗^*P* < 0.01 versus sham, *n* = 3). **(B)** Survival statistics of the sham group and WT group (χ2 = 10.457, df = 1, ^∗∗^*P* = 0.01, *n* = 20). **(C)** Prdx1 expression in the perihematomal area was detected by western blot analysis (*F* = 2.014, *t* = –3.432, ^∗∗^*P* < 0.01 versus sham, *n* = 3). **(D)** Prdx1 expression in perihematomal tissue was detected by immunohistochemistry (scale bars, 100 μm, *F* = 1.225, *t* = –7.288, ^∗∗^*P* < 0.01 versus sham, *n* = 3). **(E)** Prdx1 expression in perihematomal tissue was determined by immunofluorescence, and the percentage of positive Prdx1/NeuN, Prdx1/Ibα-1 and Prdx1/GFAP in three randomly chosen fields within the perihematomal area was counted (scale bars, 25 μm, *F* = 10.964, *t* = –11.790, ^##^*P* < 0.01 versus Prdx1/NeuN. *F* = 12.000, *t* = –60.228, ^∗∗^*P* < 0.01 versus Prdx1/NeuN, *n* = 3).

Before investigating the role of Prdx1 in ICH-induced brain injury, we first detected its expression profiles in the perihematomal tissue of ICH rat models. Our results indicated that Prdx1 mRNA level increased at 24 h, peaked at 72 h and then gradually declined (Figure 2A). Because Prdx1 expression peaked at 72h, and at same timepoint, there was a statistical difference in mortality between the two groups (Figure 2B), so we chose this timepoint for the current experiments. Using western blotting, we found that Prdx1 protein levels (Figure 2C) in model rats were significantly increased compared to the level in the sham group at 3 days after ICH. Next, we performed immunohistochemical and immunofluorescence staining, and found that in the striatum, the number of Prdx1-positive cells was significantly higher in the model rats than in the sham group (Figure 2D and Supplementary Figures S1A,B), and in the cortex, there was no significant difference in two groups (Supplementary Figures S1C,D). Furthermore, immunofluorescence staining was used to explore the cellular resource of Prdx1 in normal brain tissue and perihematomal brain tissues in the striatum (Supplementary Figure S2B), and we found that Prdx1 was mainly colocalized with GFAP^+^ astrocytes and Iba1^+^ microglia (Figure 2E and Supplementary Figure S2A). Together, these results suggest that enhanced Prdx1 expression is mainly derived from astrocytes and microglia in the striatum and may be involved in the regulation of brain injury after ICH.

### Prdx1 Suppressed ICH-Induced Apoptosis and Inflammation

**FIGURE 3:**
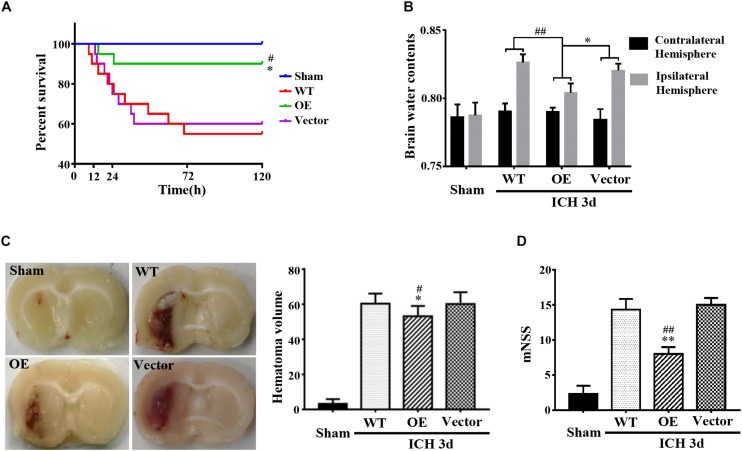
Prdx1 overexpression alleviated the symptoms of rats after ICH. **(A)** Survival statistics of the sham group, WT group, Vector group, and Prdx1-OE group (χ2 = 14.310, df = 3, ^∗^*P* < 0.05 versus Vector, ^#^*P* < 0.05 versus WT, *n* = 20). **(B)** Brain water content of the four groups (df = 3, *F* = 9.324, ^∗^*P* < 0.05 versus Vector, ^##^*P* < 0.01 versus WT, *n* = 6). **(C)** Hematoma volume of the sham group, the WT group, the Vector group, and the Prdx1-OE group (df = 3, *F* = 151.467, ^∗^*P* < 0.05 versus Vector, ^#^*P* < 0.05 versus WT, *n* = 6). **(D)** The mNSS was determined for four groups 3 days after ICH (df = 3, *F* = 75.196, ^∗∗^*P* < 0.01 versus Vector, ^##^*P* < 0.01 versus WT, *n* = 5).

**FIGURE 4:**
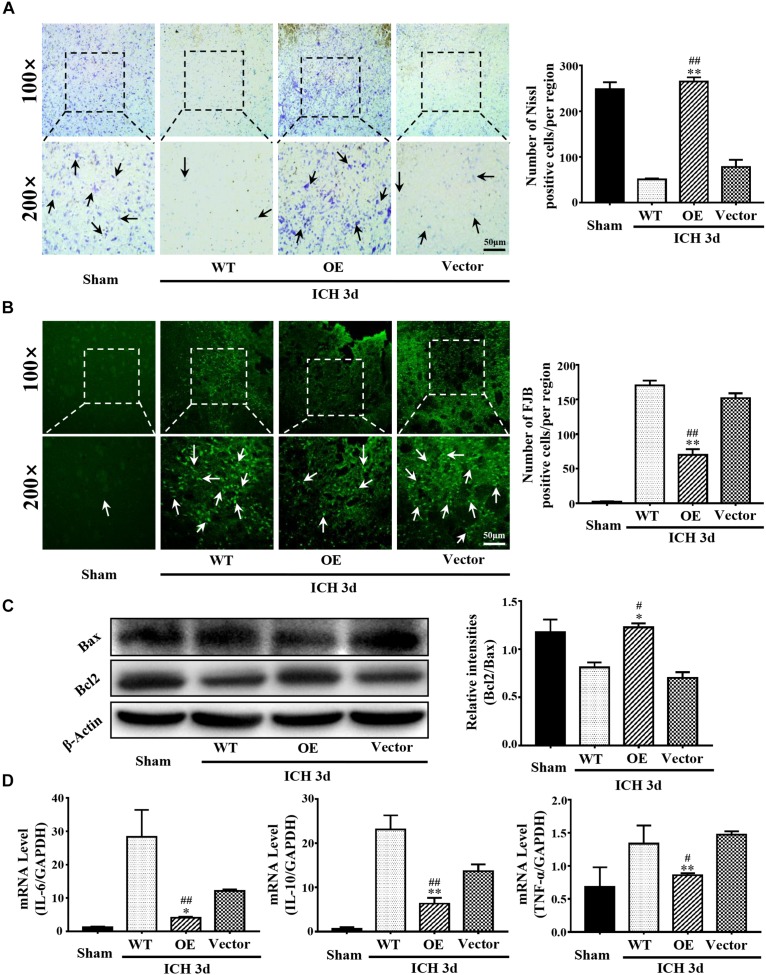
Prdx1 overexpression inhibited inflammation and apoptosis after ICH. **(A)** Representative Nissl staining in sham, WT, Prdx1-OE and Vector rats. The number of Nissl-positive cells was assessed (scale bars, 50 μm, df = 3, *F* = 174.206, ^∗∗^*P* < 0.01 versus Vector; ^##^*P* < 0.01 versus WT, *n* = 3). **(B)** Representative FJB staining in sham, WT, Prdx1-OE, and Vector rats. The number of FJB-positive cells was assessed (scale bars, 50 μm, df = 3, *F* = 435.050, ^∗∗^*P* < 0.01 versus Vector; ^##^*P* < 0.01 versus WT, *n* = 3). **(C)** Western blotting was used to detect Bcl2 and Bax expression in the four groups (df = 3, *F* = 32.759, ^∗^*P* < 0.05 versus Vector; ^#^*P* < 0.05 versus WT, *n* = 3). **(D)** Comparison of the expression of inflammatory factors in the four groups using qRT-PCR: IL-6 (df = 3, *F* = 27.046, ^∗^*P* < 0.05 versus Vector; ^##^*P* < 0.01 versus WT, *n* = 3), IL-10 (df = 3, *F* = 79.041, ^∗∗^*P* < 0.01 versus Vector, ^##^*P* < 0.01 versus WT, *n* = 3), TNF-α (df = 3, *F* = 10.274, ^∗∗^*P* < 0.01 versus Vector; ^#^*P* < 0.05 versus WT, *n* = 3).

Next, we investigated the role of Prdx1 in brain injury after ICH. Prdx1-overexpressing AAV or empty vector was injected in rats 3 weeks before ICH surgery (Supplementary Figure S3E), and the overexpression of Prdx1 in the brain was confirmed by qRT-PCR (Supplementary Figure S3C) and western blot analysis (Supplementary Figure S3D). First, we found that the mortality rate (Figure 3A) and BWC (Figure 3B) were significantly decrease in the Prdx1-OE rats compared to the WT and Vector rats after ICH. The hematoma volumes (Figure 3C) and mNSS (Figure 3D) were also significantly lower in the Prdx1-OE rats. Then, we performed Nissl staining to detect the surviving cells around the hematoma and found that the number of Nissl-positive cells in perihematomal brain tissue was higher in the Prdx1-OE group than in the WT and Vector groups (Figure 4A); in contrast, the number of damaged cells detected by FJB staining was significantly decreased in Prdx1-OE rats (Figure 4B). Furthermore, we used western blotting to investigate the expression levels of the apoptosis-related proteins Bax and Bcl2 and found that Bcl2/Bax was significantly increased in Prdx1-OE ICH rats compared with the WT and Vector groups (Figure 4C). We also assessed inflammatory factors in perihematomal brain tissue after ICH, and our results show that mRNA levels of TNF-α, IL-10, and IL-6 were markedly decreased in the Prdx1-OE group in contrast to the levels in the WT and Vector groups (Figure 4D). Together, these data indicate that Prdx1 can alleviate acute brain injury after ICH by reducing inflammation and apoptosis.

We also injected shPrdx1 AAV into rats to generate Prdx1-knockdown rats. However, most Prdx1-knockdown rats died within 3 days after ICH surgery (data not shown), and the related experiments in Prdx1-knockdown ICH rats are difficult to perform. This also illustrates the protective effect of Prdx1 in brain injury after ICH.

### Characterization of Prdx1 Binding to Target RNA *in vitro*

**FIGURE 5:**
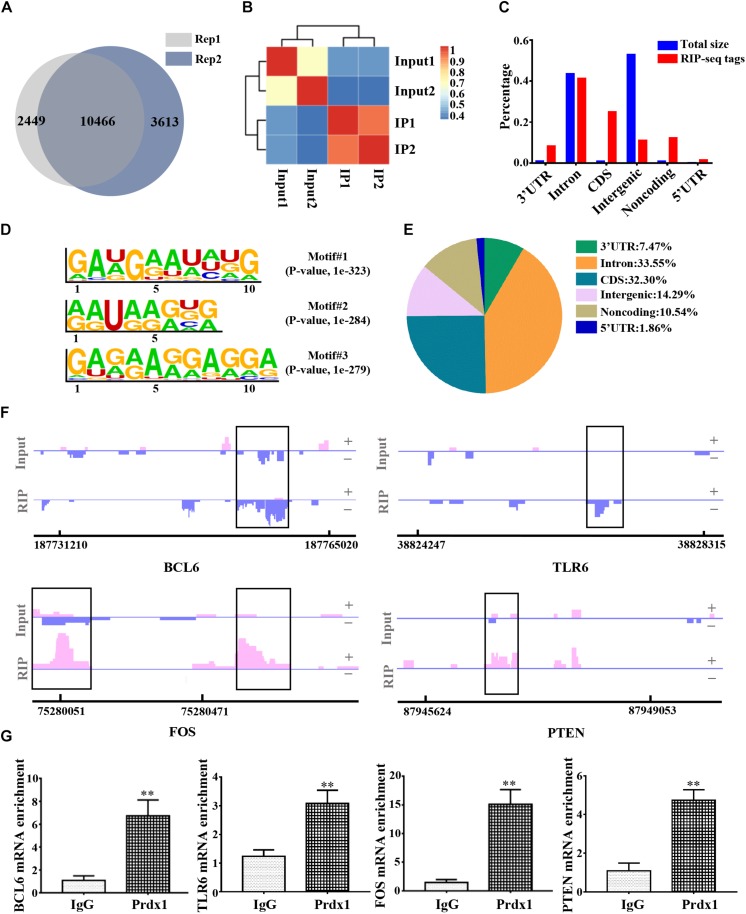
The genome-wide landscape of Prdx1 binding sites on RNA. **(A)** Venn diagram of Prdx1 RIP-seq genes from two biological replicates. **(B)** Heat map showing the correlation coefficient of two biological replicates. **(C)** The Prdx1 RIP-seq peaks are predominantly enriched in the CDS region, the 3′ UTR and the 5′ UTR. All RIP-seq peaks were classified according to their distribution on the RNA elements and compared to the genomic background. **(D)** *De novo* motif analysis identified GA repeat and GA-enriched sequences as Prdx1 binding motifs. **(E)** Prdx1 RIP-seq peak distribution proportion. **(F)** Prdx1 RIP-seq peaks are shown as track signals. The peak area is indicated by the black frame. **(G)** RIP-qPCR analysis of Prdx1 binding RNAs, IgG RIP was negative RIP control (BCL6: *F* = 3.745, *t* = –6.708, ^∗∗^*P* < 0.01 versus IgG, *n* = 3; TLR6: *F* = 1.668, *t* = –7.065, ^∗∗^*P* < 0.01 versus IgG, *n* = 3; FOS: *F* = 5.224, *t* = –10.432, ^∗∗^*P* < 0.01 versus IgG, *n* = 3; PTEN: *F* = 0.000, *t* = –6.998, ^∗∗^*P* < 0.01 versus IgG, *n* = 3).

To further delineate the underlying mechanism of Prdx1 in reducing inflammation and apoptosis, we used RNA immunoprecipitation coupled with next-generation sequencing (RIP-seq) to identify all RNAs that interact with Prdx1. We chose HeLa cells for these experiments for the following reasons: studies have shown that HeLa cells are model cells for studying RBPs (D’Amico et al., 2019; Loughlin et al., 2019; Wilbertz et al., 2019) and HeLa cells are good for gene regulation studies in the central nervous system (Chu et al., 2013). In addition, Prdx1 also shows anti-apoptotic and anti-inflammatory effects in HeLa cells (Nassour et al., 2016) (Supplementary Figure S4A), suggesting that Prdx1 may play a similar role in the central nervous system. First, we tested the consistency of two biological replicates of Prdx1 RIP-seq data and found 10466 consensus genes, showing that our results were reproducible (Figure 5A,B and Supplementary Data 1). Next, we analyzed the regions of the Prdx1-bound RNAs. By comparison with the whole genome, we found the regions where the Prdx1-bound RNAs were mainly concentrated in the coding sequence (CDS) region, the 5′ UTR and the 3′ UTR (Figures 5C,E). Furthermore, *de novo* motif analysis showed that the Prdx1-bound motif was mainly in the AG-enriched region (Figure 5D), and GO Ontology (GO) analysis of Prdx1-bound RNAs revealed significantly enriched terms in RNA/mRNA processing and splicing (Supplementary Figure S4B), which is consistent with our prediction. Our Prdx1 RIP-seq results revealed that Prdx1 bound to mRNA transcripts was associated with the inflammatory response (BCL6 and TLR6) and the apoptotic process (PTEN and FOS) (Figure 5F), consistent with the RIP-qPCR result for these mRNAs in astrocytes (Figure 5G), suggesting that Prdx1 plays a role in inflammation and apoptosis.

### Prdx1 Affects Inflammation- and Apoptosis-Related mRNA Stability

**FIGURE 6:**
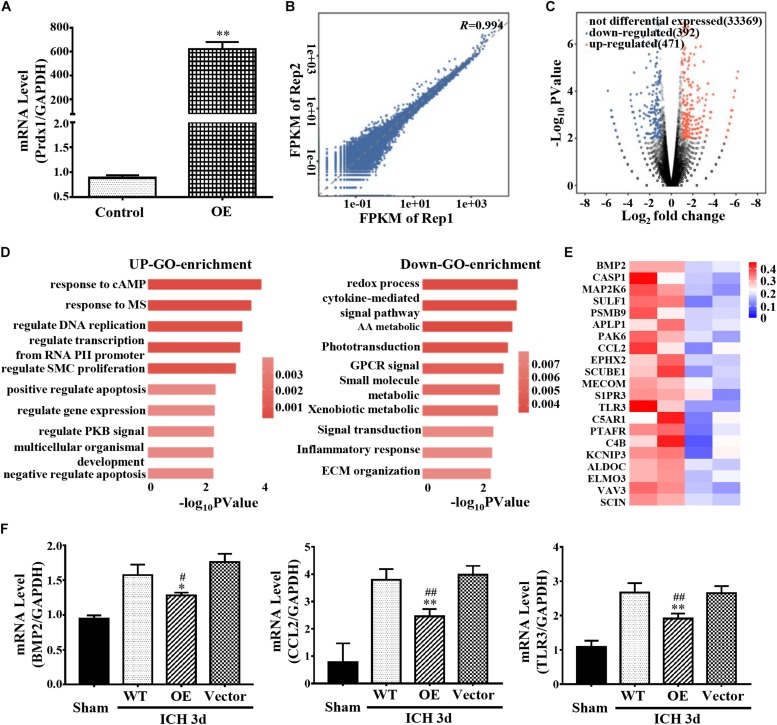
Prdx1 affects inflammation- and apoptosis-related mRNA stability. **(A)** Prdx1 expression in control HeLa cells and in the Prdx1-OE group was detected by qRT-PCR (*F* = 8.494, *t* = 40.635, ^∗∗^*P* < 0.01 versus Control). **(B)** Scatter plots and correlation coefficients of two biological replicates of RNA-seq. **(C)** Volcano map: red dot indicates a gene that is upregulated after Prdx1 overexpression, black dot indicates a gene with no significant change, and blue dot indicates a downregulated gene. **(D)** Gene ontology (GO) analysis of Prdx1-dependent DEGs. Significantly enriched GO terms of genes. The *x*-axis indicates the enrichment *P*-value on a −log10 scale; the *y*-axis indicates terms. **(E)** Heat map showing that apoptosis- and inflammation-related genes were significantly decreased after Prdx1 was upregulated in HeLa cells. **(F)** qRT-PCR was performed in four groups 3 days after ICH (BMP2: *F* = 8.949, df = 3, ^∗^*P* < 0.05 versus Vector, ^#^*P* < 0.05 versus WT, *n* = 3; CCL2: *F* = 33.103, df = 3, ^∗∗^*P* < 0.01 versus Vector, ^##^*P* < 0.01 versus WT, *n* = 3; TLR3: *F* = 39.330, df = 3, ^∗∗^*P* < 0.01 versus Vector, ^##^*P* < 0.01 versus WT, *n* = 3).

Prdx1 mainly binds to the 3′ UTR, CDS, and 5′ UTR of the RNAs, indicating that its function may be related to the stability of RNA and AS (Mayr, 2016; Yee et al., 2019). To obtain a comprehensive view of Prdx1-dependent DEGs, control or Prdx1-overexpressing vector was transfected into HeLa cells (Figure 6A and Supplementary Figure S5A) and RNA-seq was performed. The RNA-seq data showed good reproducibility (*R* = 0.994, Figure 6B). A total of 863 Prdx1-dependent DEGs were identified; among them, 471 were upregulated genes, and 392 were downregulated genes (Figure 6C, Supplementary Figure S5B, and Supplementary Data 2). Next, we performed GO analysis on these DEGs and found that these genes were mainly associated with redox reactions, cytokine pathways, and inflammatory responses, as well as with apoptosis and DNA repair (Figure 6D). Further, we screened for genes involved in inflammation and apoptosis in these DEGs (Figure 6E and Supplementary Data 3), verified their changing profiles using qRT-PCR, and found similar trends of these mRNAs in ICH rats when Prdx1 overexpression was compared with RNA-seq data in HeLa cells (Figure 6F and Supplementary Figure S5C).

**FIGURE 7:**
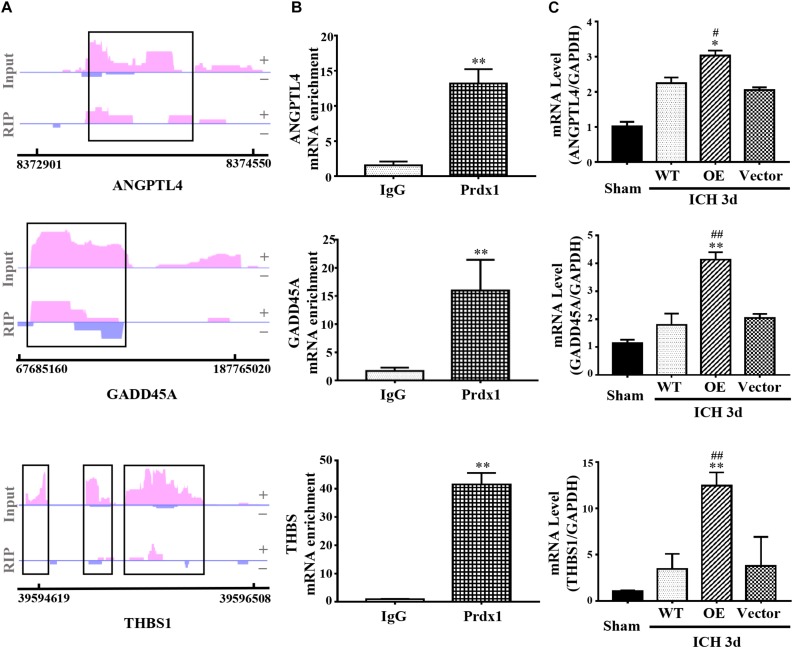
Prdx1 plays similar roles *in vitro* and *in vivo*. **(A)** Prdx1 RIP-seq peaks are shown as track signals of ANGPTL4, GADD45A and THBS1. The peak area is indicated by the black frame. **(B)** RIP-qPCR analysis of Prdx1 binding RNAs in astrocytes with IgG RIP as a negative RIP control (ANGPTL4: *F* = 4.297, *t* = –13.551, ^∗∗^*P* < 0.01 versus IgG, *n* = 3; GADD45A: *F* = 12.277, *t* = –8.909, ^∗∗^*P* < 0.01 versus IgG, *n* = 3; THBS: *F* = 4.072, *t* = –20.619, ^∗∗^*P* < 0.01 versus IgG, *n* = 3). **(C)** qRT-PCR was performed in four groups of ANGPTL4, GADD45A, and THBS1 mRNA (ANGPTL4: *F* = 87.049, df = 3, ^∗^*P* < 0.05 versus Vector, ^#^*P* < 0.05 versus WT, *n* = 3; GADD45A: *F* = 73.252, df = 3, ^∗∗^*P* < 0.01 versus Vector, ^##^*P* < 0.01 versus WT, *n* = 3; THBS: *F* = 20.553, df = 3, ^∗∗^*P* < 0.01 versus Vector, ^##^*P* < 0.01 versus WT, *n* = 3).

Coupling RIP-seq and RNA-seq, we found that Prdx1 can combine with inflammation- and apoptosis-related RNAs, such as ANGPTL4, GADD45A, and THBS1, and cause differential expression of these mRNAs in HeLa cells (Figure 7A and Supplementary Data 2). We validated these results in astrocytes and ICH models, and found that Prdx1 also combined with these mRNAs in astrocytes (Figure 7B); similar trends of these mRNA levels were confirmed in the rat ICH model (Figure 7C).

## Discussion

RNA-binding protein-mediated posttranscriptional regulation plays an important role in the pathophysiological processes of diseases (Wurth et al., 2016; Bar-Ziv et al., 2019; Baser et al., 2019). Recent studies have shown that posttranscriptional regulation is also associated with ICH (Dykstra-Aiello et al., 2015; Stamova et al., 2019). However, what roles RBPs play in ICH is still largely unknown. In this study, we found that Prdx1, an RBP, was significantly increased in astrocytes and microglia in response to brain injury after ICH, that is regulated inflammation- and apoptosis-related mRNA stability, and that is reduced inflammation- and apoptosis-related molecular production and release, thereby inhibiting the inflammatory response and reducing apoptosis after ICH, suggesting that Prdx1 could serve as an improved therapeutic target to inhibit both inflammation and apoptosis after ICH.

In this study, we first found that Prdx1 expression in perihematomal brain tissues was markedly increased, which in line with previous studies (Nakaso et al., 2000; Liu et al., 2014). Immunofluorescent staining revealed that elevated Prdx1 was mainly expressed in astrocytes and microglia but not in neurons in the striatum of ICH rats. Interestingly, we also found that Prdx1 colocalized with NeuN^+^ neurons in the cortex (Supplementary Figure S6), differential cellular localization of Prdx1 indicate that Prdx1 expression in the CNS may exert functional diversity. In addition, our results show that Prdx1 can reduce the hematoma volume after ICH, its mechanism needs further study, we speculate that Prdx1 inhibits the release of inflammatory factors such as TNF-α, thereby promoting the expression of CD36 and causing the absorption of hematomas.

While previous studies investigated the roles of Prdx1 in other diseases, our study is the first to demonstrate that Prdx1 can reduce inflammation and apoptosis after ICH. Liu et al. showed that Prdx1 exacerbate brain injury after ICH (Liu et al., 2016). However, the study did not use Prdx1 overexpression or interference models to verify the effect of Prdx1 after ICH, but only inferred its role in ICH based on the function of Prdx1 in macrophages. In fact, Prdx1 performs different and even opposite functions in different cell types, thus the study failing to fully elucidate the role and mechanisms of Prdx1 in ICH. In our study, we first overexpressed Prdx1 in rat brain and established ICH model, found that the symptoms of Prdx1-OE group was significantly alleviated compare with WT or Vector group, our study provided direct evidence of its role in ICH.

However, the roles of Prdx1 in regulating inflammation and apoptosis are currently controversial. For example, Liu et al. reported that Prdx1 can promote the release of inflammatory mediators by activating macrophages (Liu et al., 2014; He et al., 2019), and Riddell et al. declare Prdx1 could activate the TLR4/MyD88 signaling pathway (Riddell et al., 2010, 2012), thereby enhancing inflammation and apoptosis. However, Lu et al. showed that Prdx1 reduces the inflammatory response by inhibiting the activation of NF-κB and oxidative stress (Min et al., 2018; Lu et al., 2019), and studies have also shown that Prdx1 inhibits the activity of the ubiquitin ligase TRAF6 (Min et al., 2018), thereby producing an anti-inflammatory effect. Our data suggests that Prdx1 inhibits inflammation and apoptosis by regulating the posttranscriptional regulation of inflammation- and apoptosis-related mRNAs, and thus provides new evidence and insights into the anti-inflammatory effects of Prdx1.

Kim et al. (2012) first reported that Prdx1 can act as an RBP to bind RNAs, which has also been demonstrated in a number of recent studies (Baltz et al., 2012; Castello et al., 2012; Kwon et al., 2013; Sebestyén et al., 2016). However, it is still not clear what kinds of RNAs Prdx1 can combine with and what the effects of such couplings are. In the current study, we first investigated the properties of Prdx1 as an RBP from a genome-wide perspective and found that Prdx1 can regulate mRNA stability because Prdx1 is mainly combined with the CDS region, the 3′ UTR and 5′ UTR in AG-enriched RNA motifs. For example, we found that Prdx1 can combine with ANGPTL4, GADD45A, and THBS1 mRNAs, and it upregulated these mRNAs when Prdx1 was overexpressed. Previous studies showed that ANGPTL4 can significantly inhibit the inflammatory response (Guo et al., 2015) and alleviate neurological deficits and cerebral edema after ICH (Qiu et al., 2018). GADD45A is a DNA damage-inducing protein and has been shown to inhibit oxidative stress and inflammatory responses (Tanaka et al., 2017; Li F. H. et al., 2018). THBS1 is also an anti-inflammatory molecule (Cursiefen et al., 2011) and accelerates synaptogenesis (Xu et al., 2010). These earlier findings, together with our current results, demonstrate the anti-inflammatory and anti-apoptotic effects of Prdx1 and suggest Prdx1 as an improved therapeutic target to inhibit both inflammation and apoptosis after ICH.

## Data Availability Statement

The datasets generated for this study can be found in the GEO, GSE134505.

## Ethics Statement

The animal study was reviewed and approved by the Animal Management Committee of the Third Military Medical University.

## Author Contributions

Q-WY and X-YX designed the research presented in this manuscript. G-QY and J-CH conducted the cell cultures and qRT-PCR as well as the histological and behavioral analyses. J-JY, C-XG, and QZ performed the western blot and the RIP-seq. QX, L-XX, and RC built the ICH rat model. Z-MQ, KZ, and G-HJ performed the RNA-seq. RX and QC conducted the statistical analysis.

## Conflict of Interest

The authors declare that this study received advice from the Wuhan Life Beauty Technology Co., Ltd. The company was not involved in the study design, collection, analysis, interpretation of data, the writing of this article or the decision to submit it for publication.
