# Supplementary material for: Prdx1 Reduces Intracerebral Hemorrhage-Induced Brain Injury via Targeting Inflammation- and Apoptosis-Related mRNA Stability
Source: Front Neurosci. 2020 Mar 10;14:181. doi: 10.3389/fnins.2020.00181 (PMC7076121; doi:10.3389/fnins.2020.00181)
Supplement: Supplementary file 1 [file Table_1.docx]

**Supplementary Table 1.** **Primers used for quantitative real-time PCR**

| Primers | Forward | Reverse |
| --- | --- | --- |
| Rat IL-10 | 5’-AAAGCAAGGCAGTGGAGCAG-3’ | 5’-AGTAGATGCCGGGTGGTTCA-3’ |
| Rat IL-6 | 5’-AGGAGTGGCTAAGGACCAAGACC-3’ | 5’-TGCCGAGTAGACCTCATAGTGACC-3’ |
| Rat TNFα | 5’-GCATGATCCGAGATGTGGAACTGG-3’ | 5’-CGCCACGAGCAGGAATGAGAAG-3’ |
| Rat GAPDH | 5’-TCCCTCAAGATTGTCAGCAA-3’ | 5’-AGATCCACAACGGATACATT-3’ |
| Rat Prdx1 | 5’-CGCACCATTGCTCAGGAT-3’ | 5’-AGCGGCCAACAGGAAGAT-3’ |
| Rat BMP2 | 5’-CAGCGGAAGCGTCTTAAGTCCAG-3’ | 5’-GGCATGGTTGGTGGAGTTCAGG-3’ |
| Rat TLR3 | 5’-TTCGTCCTCCTCTTGAACAATGCC-3’ | 5’-CAAGCGAGGTGAGGTTGGTCTG-3’ |
| Rat CCL2 | 5’-CTATGCAGGTCTCTGTCACGCTTC-3’ | 5’-CCAGTGAATGAGTAGCAGCAGGTG  -3’ |
| Rat S1PR3 | 5’-GGTTGGTGTGCGGCTGTCTG-3’ | 5’-AGAGTGGCTGCTGCTGTTGTTAC-3’ |
| Rat C5AR1 | 5’-GGATGGCTTGCGGAGTAACCTG-3’ | 5’-TTAAGAGTGAGCAGAGGCAACACG-3’ |
| Rat THBS1 | 5’-GGCTTCATCTTCCTGGCTTCCTTG-3’ | 5’-GCTTCCTCCACTGACACCACTTG-3’ |
| Rat GADD45A | 5’-CACCATAACTGTCGGCGTGTACG-3’ | 5’-CATCTTCATCCGCAGCCAGCAG-3’ |
| Rat ANGPTL4 | 5’-CAGCAGCCTCCTAGCCTCCTC-3’ | 5’-TGTCCACGAGACTCCAGATAGCC-3’ |
| Human GAPDH | 5‘-GAAGGTGAAGGTCGGAGTC-3’ | 5’-GAAGATGGTGATGGGATTTC -3’ |
| Human Prdx1 | 5’-AAGGCTGATGAAGGCATCTCGTTC-3’ | 5’-CATCCACAGAGCGGCCAACAG-3’ |
| Rat BCL6 | 5’-TGGACTGTGAAGCAAGGCATTGG-3’ | 5’-CGGAGGCGGTTAAGGTTGAGAAG-3’ |
| Rat PTEN | 5’-TTGAAGACCATAACCCACCACAGC-3’ | 5’-CATTACACCAGTCCGTCCTTTCCC-3’ |
| Rat TLR6 | 5’-TCCCACCCCTCCTCTGATTTCTTC-3’ | 5’-CCACCACTTCTCTTGATGCCTGAC-3’ |
| Rat FOS | 5’-AGACCATGTCAGGCGGCAGAG-3’ | 5’-GTCAGCTCCCTCCTCCGATTCC-3’ |
