# Supplementary material for: Prdx1 Reduces Intracerebral Hemorrhage-Induced Brain Injury via Targeting Inflammation- and Apoptosis-Related mRNA Stability
Source: Front Neurosci. 2020 Mar 10;14:181. doi: 10.3389/fnins.2020.00181 (PMC7076121; doi:10.3389/fnins.2020.00181)
Supplement: Supplementary file 2 [file Table_2.docx]

**Supplementary Table 2.** **Modified Neurological Severity Scores(mNSS)**

| Raising rat by tail (normal =0; maximum=3) | Score  (3) |
| --- | --- |
| Flexion of forelimb  Flexion of hindlimb  Head moved>10^o^ to vertical axis within 30S | 1  1  1 |
| Placing rat on floor (normal=0; maximum=3) | (3) |
| Normal walk  Inability to walk straight  Circling toward paretic side  Falls down to paretic side | 0  1  2  3 |
| Sensory tests (normal=0; maximum=6) | (6) |
| Balances with steady posture  Grasps side of beam  Hugs beam and 1 limb falls down from beam  Hugs beam and 2 limbs fall down from beam, or spins on beam(>60s)  Attempts to balance on beam but falls off(>40s)  Attempts to balance on beam but falls off(>20s)  Falls off; no attempt to balance or hang on to beam(<20s) | 0  1  2  3  4  5  6 |
| Reflex absence and abnormal movements | (4) |
| Pinna reflex (head shake when auditory meatus ids touched)  Corneal reflex (eye blink when cornea is lightly touched with cotton)  Startle reflex (motor response to a brief noise)  Seizures, myoclonus, myodystony | 1  1  1  1 |
