# Supplementary material for: Prdx1 Reduces Intracerebral Hemorrhage-Induced Brain Injury via Targeting Inflammation- and Apoptosis-Related mRNA Stability
Source: Front Neurosci. 2020 Mar 10;14:181. doi: 10.3389/fnins.2020.00181 (PMC7076121; doi:10.3389/fnins.2020.00181)
Supplement: Supplementary file 3 [file Data_Sheet_1.docx]

**Supplementary Figure 1**


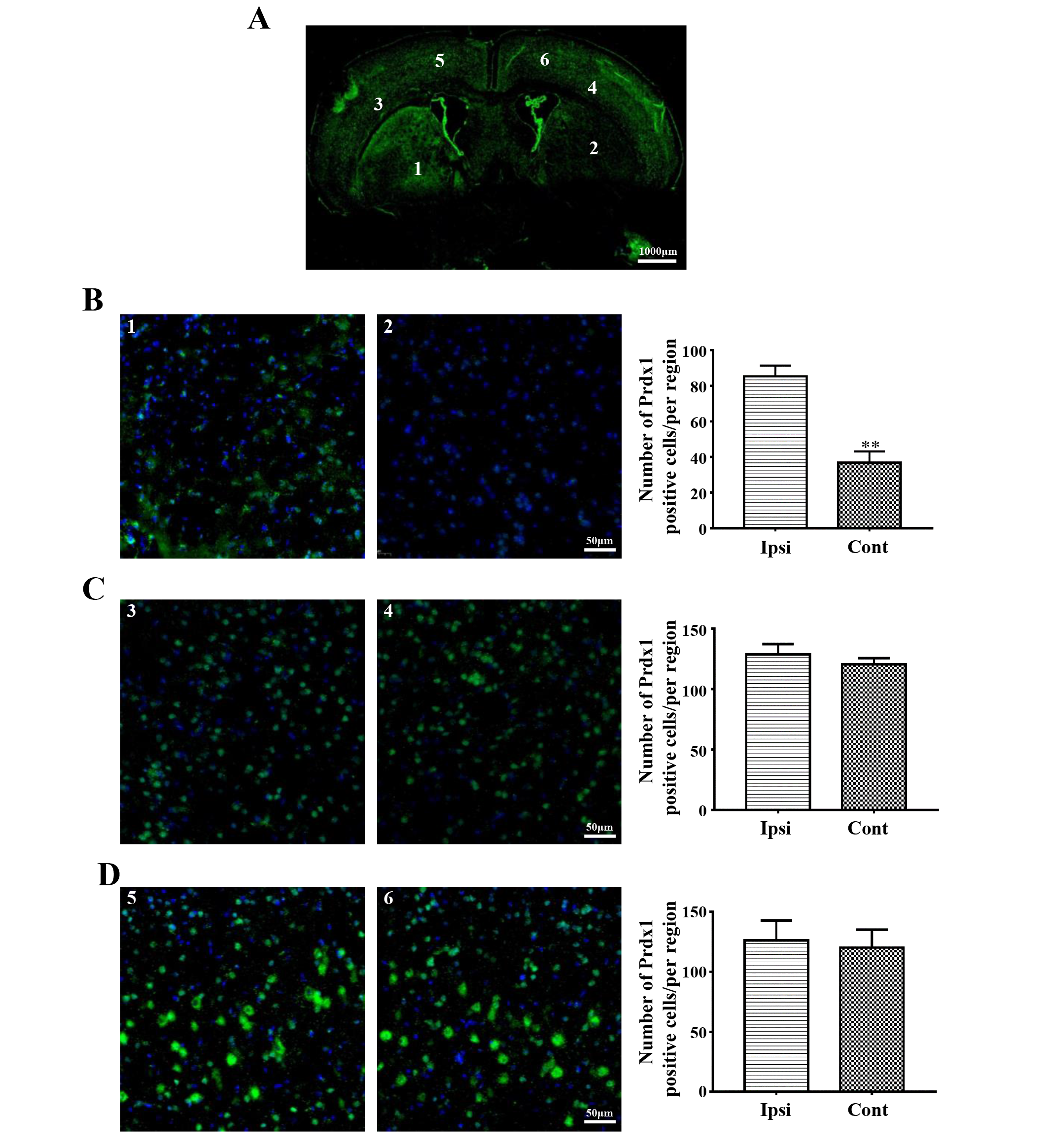


**Supplementary Figure 1.** **Prdx1 expression was significantly increased in striatum after ICH.** (A) Prdx1 expression was detected by immunofluorescence (scale bars, 1000 μm). (B) Compare the number of Prdx1 positive cells in ipsilateral and contralateral striatum (scale bars, 50 μm, F = 0.007, t = 9.504, **P < 0.01 versus ipsilateral, n = 3). (C) Compare the number of Prdx1 positive cells in ipsilateral and contralateral cortex near the striatum (scale bars, 50 μm, F = 1.216, t = 1.537, P > 0.05 versus ipsilateral, n = 3). (D) Compare the number of Prdx1 positive cells in ipsilateral and contralateral cortex (scale bars, 50 μm, F = 0.059, t = 0.468, P > 0.05 versus ipsilateral, n = 3).

**Supplementary Figure 2**

**
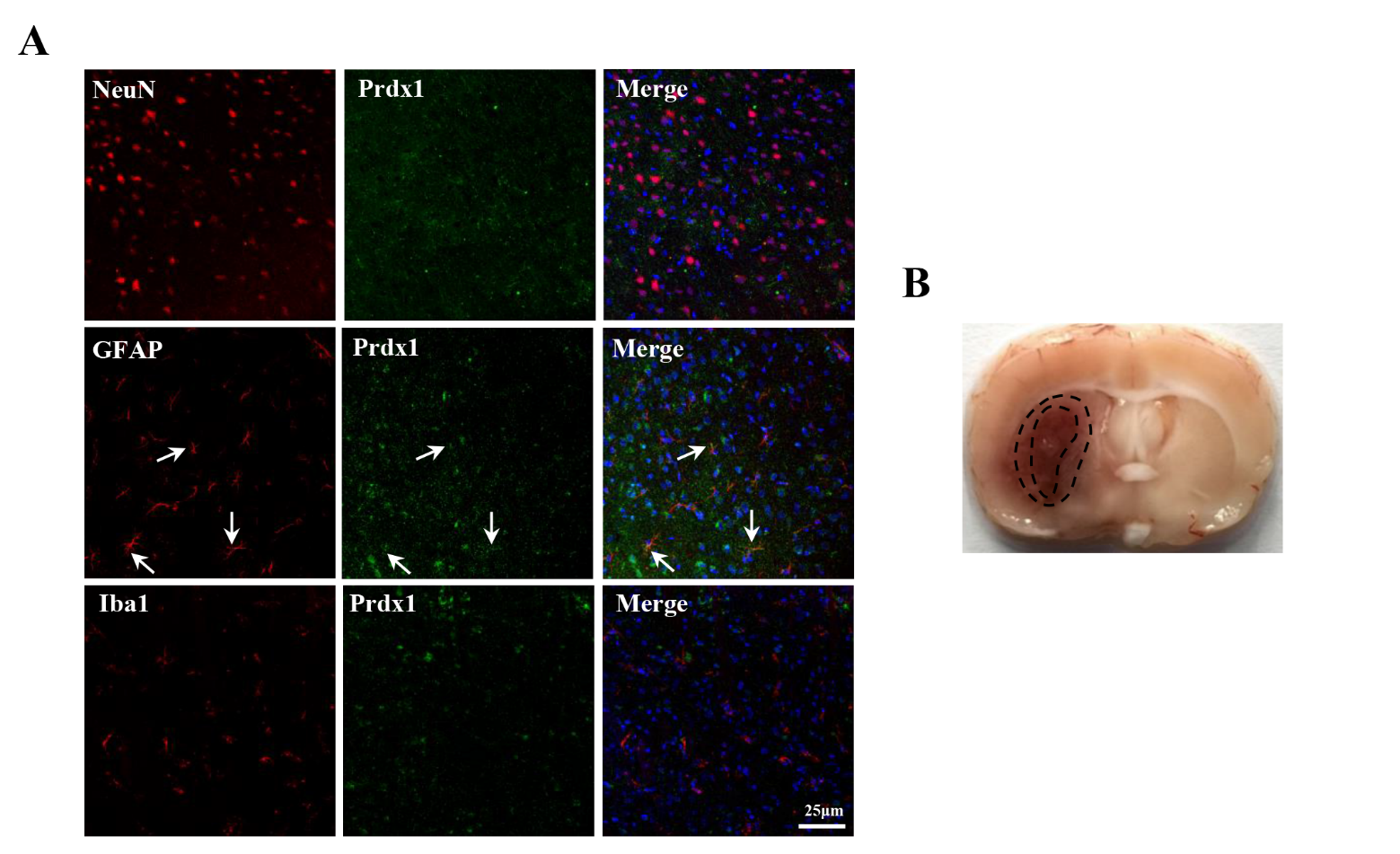
**

**Supplementary Figure 2. Prdx1 expression in nomal brain tissue was detected by immunofluorescence.** (A) Prdx1 expression in striatum tissue was detected by immunofluorescence, double positive cells are indicated by arrows. (B) The part inside the two dotted lines is the perihematomal tissue.

**Supplementary Figure 3**


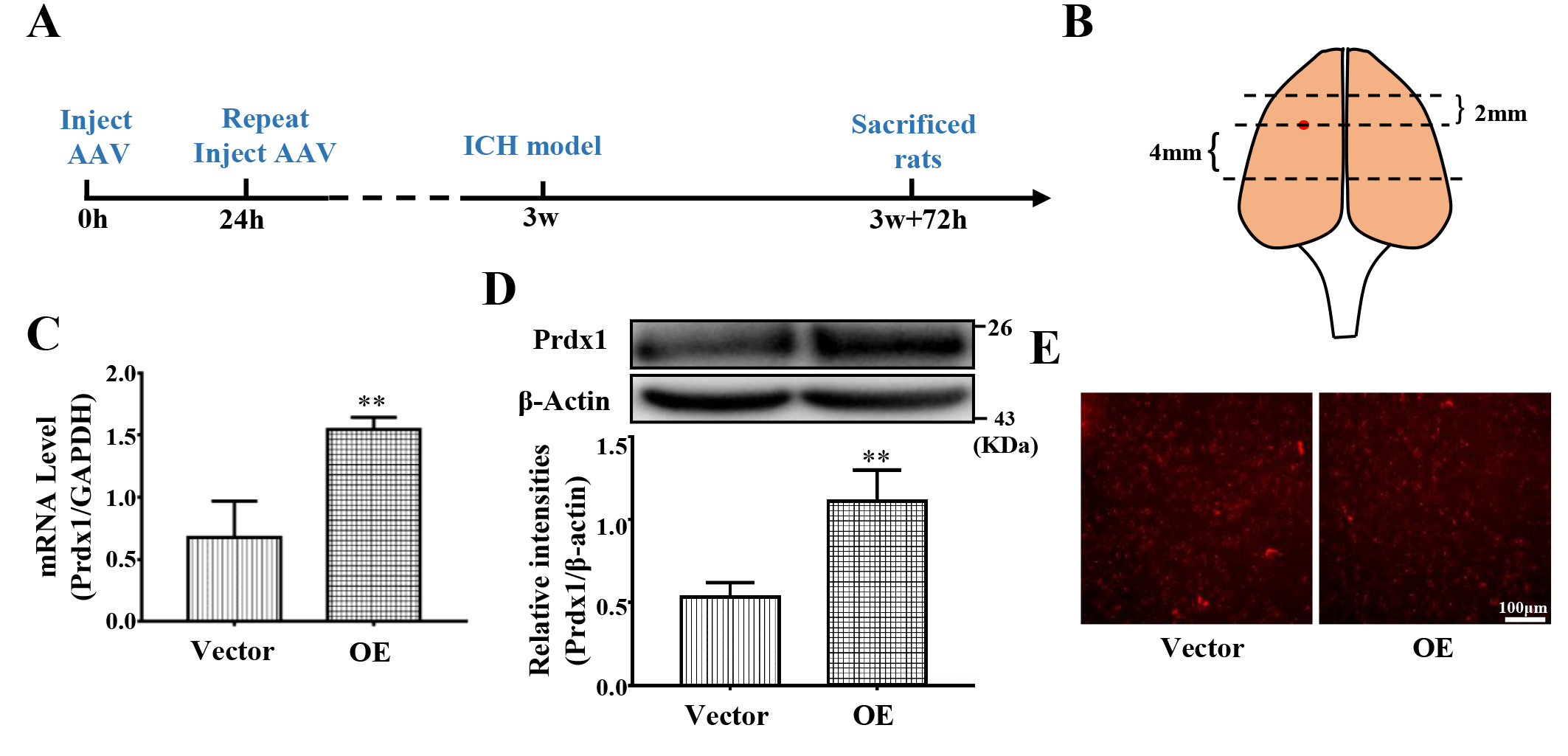


**Supplementary Figure 3. Establishment of the Prdx1 overexpression model.** (A) A chronological table showing the time point of intervention in rats. (B) Schematic diagram of perihematomal tissue. Prdx1 expression in the Vector group and Prdx1-OE group was detected by (C) qRT-PCR (F = 2.532, t = -4.859, **P < 0.01 versus Vector, n = 3), (D) Western blotting (F = 6.231, t = -4.806, **P < 0.01 versus Vector, n = 3) and (E) fluorescence (Scale bars, 100 μm).

**Supplementary Figure 4**


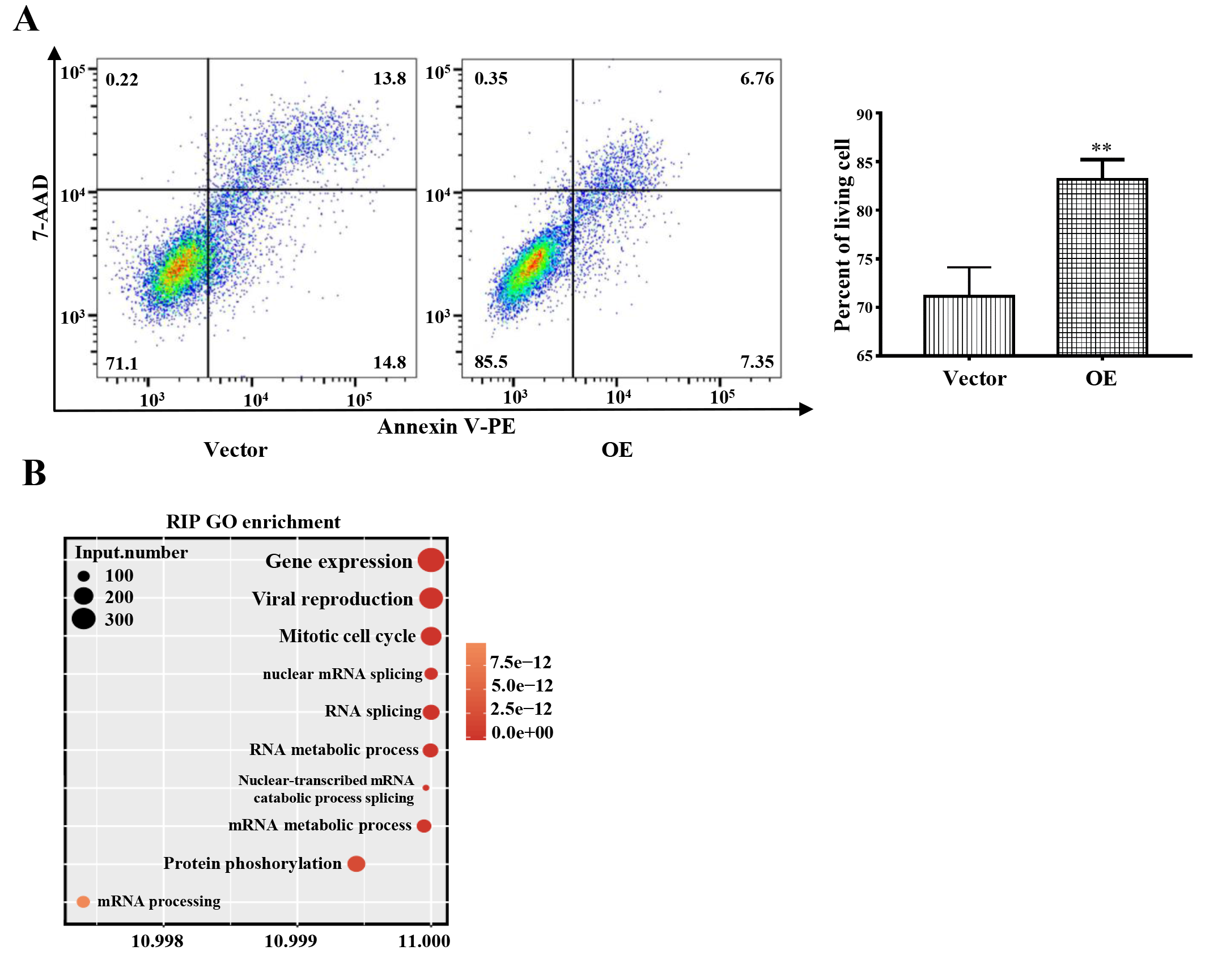


**Supplementary Figure 4. Anti-apoptotic effect and the genome-wide landscape of Prdx1 binding sites on RNA.** (A) Flow cytometry was used to detect the proportion of living cells (F = 0.727, t = -5.376, **P < 0.01 versus WT, n = 3). (B) Gene Ontology (GO) analysis of the genes that Prdx1 binds.

**Supplementary Figure 5**


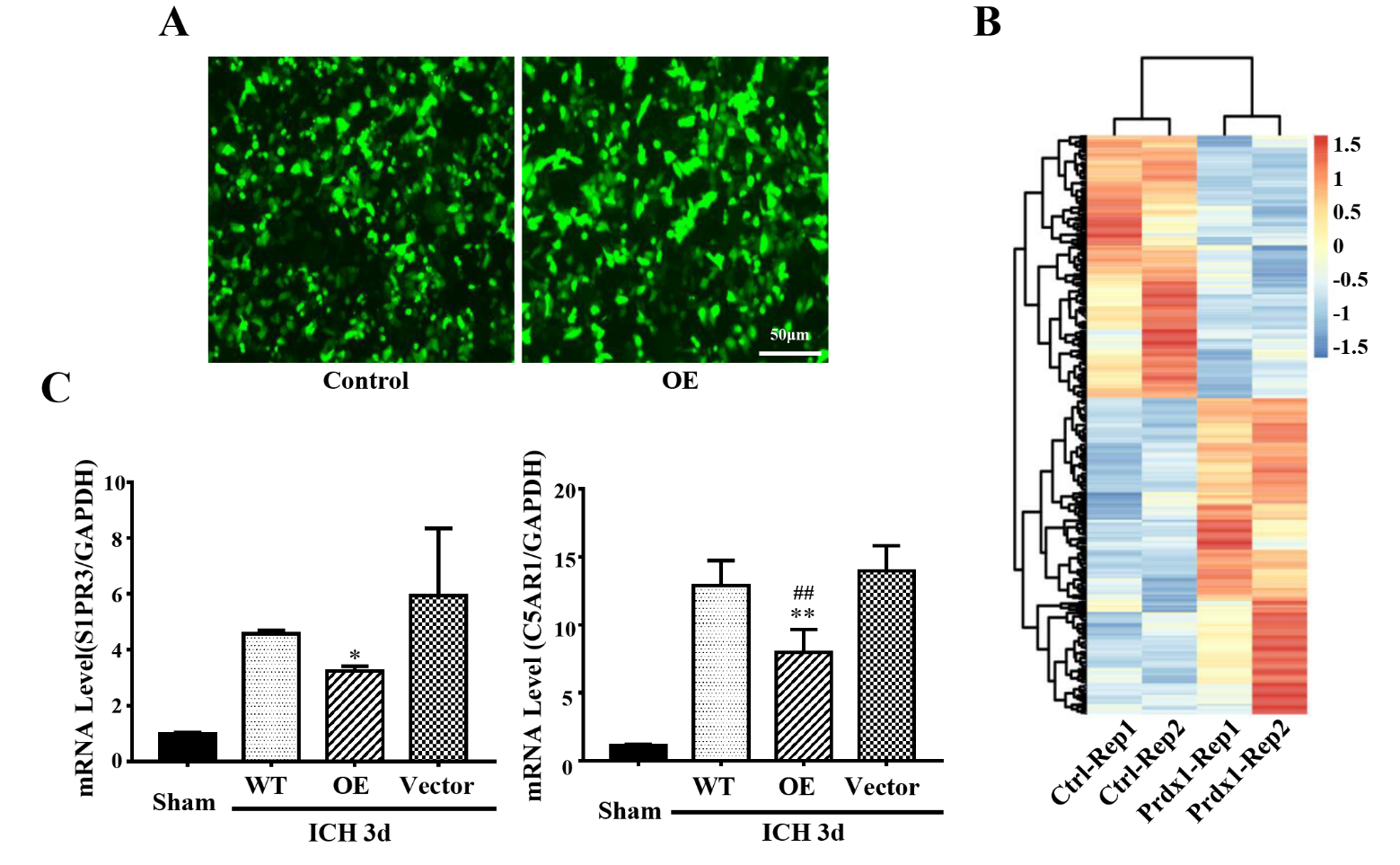


**Supplementary Figure 5. Prdx1 affects inflammation- and apoptosis-related mRNA stability.** (A) GFP indicates Control or Prdx1-overexpressing vector was successfully tranfected into Hela cells. (B) Heat map showing differentially expressed genes after prdx1 upregulated in HeLa cells. (C) qRT-PCR was performed in four groups 3d after ICH (S1PR3: df = 3, F = 8.725, *P < 0.05 versus Vector; C5AR1: df = 3, F = 55.678,**P < 0.01 versus Vector, ##P < 0.01 versus WT, n = 3).

**Supplementary Figure 6**


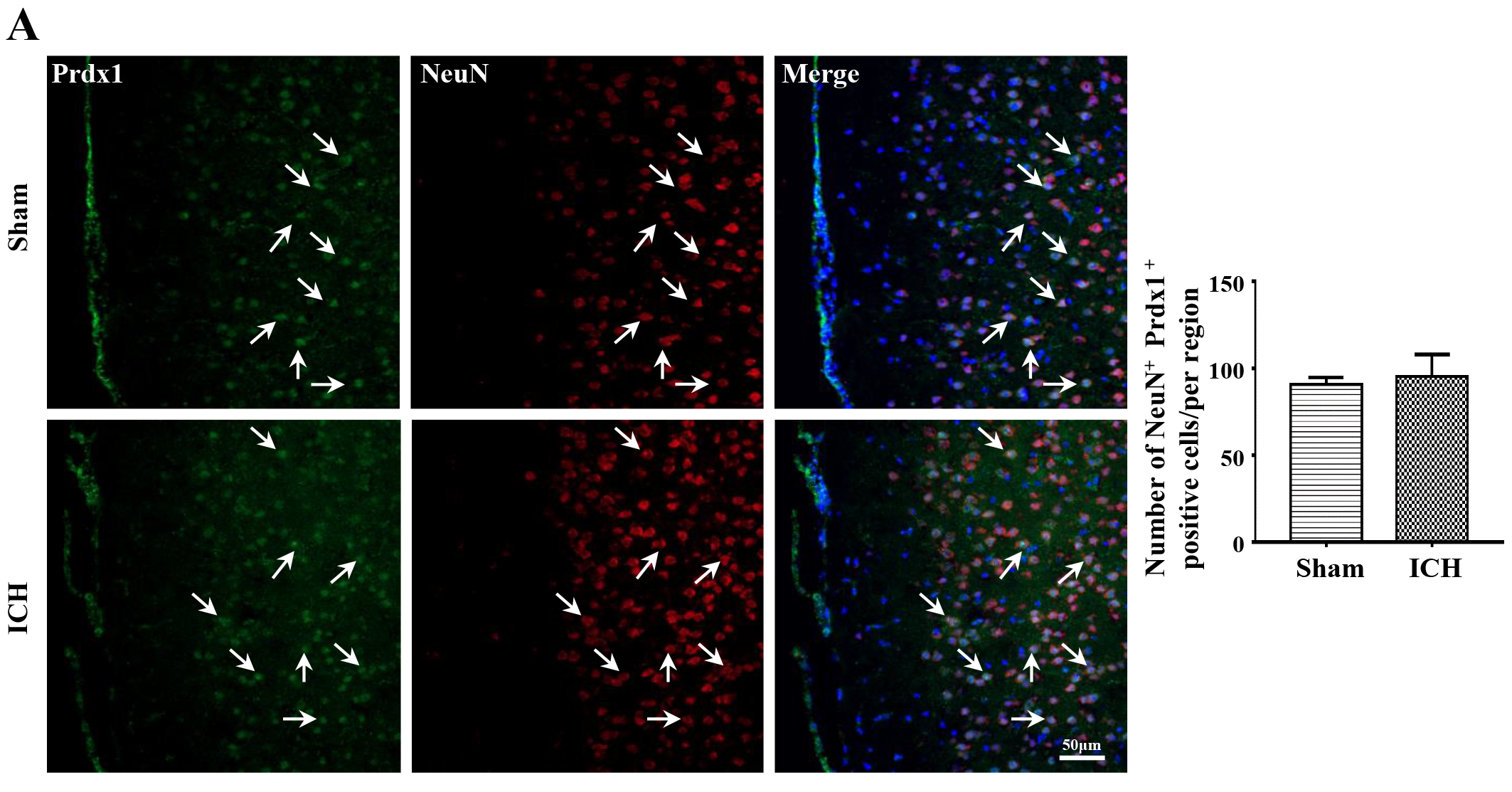


**Supplementary Figure 6. Prdx1 expression in neuron in the cortex.** (A) Compare the number of NeuN^+^ Prdx1^+^ cells in the cortex in two groups (scale bars, 50 μm, F = 2.931, t = -0.608, P > 0.05 versus Sham, n = 3).
